# Postchemotherapy Histopathological Evaluation of Ovarian Carcinoma: A 40-Case Study

**DOI:** 10.1155/2015/197871

**Published:** 2015-01-21

**Authors:** Kanwardeep Kaur Tiwana, Sarita Nibhoria, Manmeet Kaur, Tanvi Monga, Ratika Gupta

**Affiliations:** ^1^Department of Pathology, GGS Medical College, Faridkot, Punjab 151203, India; ^2^Department of Radiotherapy, GGS Medical College, Faridkot 151203, India

## Abstract

Ovarian carcinomas are conventionally treated with primary debulking surgery followed by chemotherapy. Nowadays neoadjuvant chemotherapy followed by surgery is an upcoming treatment modality for ovarian carcinoma. This study highlights the histopathological changes observed after neoadjuvant chemotherapy. Present study is a 40-case study stressing five histological parameters: residual tumour, fibrosis, necrosis, inflammation, and psammoma bodies. All these parameters carry prognostic significance and they are easily reproducible. Fleiss kappa statistics were used to measure intraobserver agreement between pathologists which was found to be substantial to almost perfect with *κ* ranging between 0.621 and 1.00. This study highlights easily reproducible parameters and their incorporation in histopathology report, thus helping in patient management.

## 1. Introduction 

Ovarian carcinoma is one of the most common female malignancies worldwide and is the second most common gynecological cancer after cervical cancer [1, 2]. Among women in United States ovarian cancer is the eighth most common cancer and the fifth leading cause of cancer deaths [3]. However it causes more deaths than any other cancer of female reproductive system [3]. In contrast ovary is second leading site among gynecological malignancies in India [1]. The conventional treatment modality for advanced ovarian carcinoma is primary cytoreduction surgery followed by chemotherapy. But the new approach is neoadjuvant platinum based chemotherapy followed by interval debulking surgery. National Comprehensive Cancer Network guidelines for 2012 recommend the approach as this treatment modality reduces surgical morbidity with equivalent survival times [4]. Due to this new approach various morphological changes are seen in the tumour cells and stroma. Various changes seen are nuclear enlargement, hyperchromasia, bizarre nuclei, nuclear clumping, cytoplasmic eosinophilia, vacuolization, and foamy cell change. The stromal changes observed are fibrosis, inflammation, necrosis, and dystrophic calcification including presence of psammoma bodies. Minimal residual disease is defined by presence of residual tumour ≤1 cm and is one of the best predictive good prognostic factors [5–7]. There are two aims of the study. First is to highlight detailed postchemotherapy histopathological parameters, particularly residual tumour, inflammation, necrosis, fibrosis, and psammoma bodies, on haematoxylin and eosin stained sections [4, 7, 8]. Secondly is to stress upon the fact that there is need to change reporting formats of ovarian carcinoma incorporating these histopathological changes so that they can provide an extra tool to optimize patient management and care.

## 2. Materials and Methods

This is a three-year study conducted in the Department of Pathology, Guru Gobind Singh Medical College, Faridkot, Punjab, during the period of January 2010 to December 2013 consisting of 40 cases of ovarian carcinoma that had been treated with preoperative chemotherapy. Inclusion criteria used were cases which were Ultrasound guided FNAC (fine needle aspiration cytology) proven, raised CA125 and those having malignant effusion. Patients having any one of the abovementioned or combination of any of these three were chosen for chemotherapy followed by debulking surgery. These patients were given three to four rounds of paclitaxel and carboplatin combination chemotherapy followed by surgery. Detailed histopathological evaluation was carried out especially for chemotherapy related changes. The histopathological changes were documented separately by three pathologists having experience in their field and evaluation was done on H & E (haematoxylin and eosin) sections. Five parameters were considered for assessment, that is, fibrosis, necrosis, residual tumour, inflammation, and psammoma bodies. Fibrosis was scored as mild (1+), moderate (2+), and severe (3+). Necrosis was scored as absent (0), 1%–50% (1+), and present >50% (2+). Residual tumour was scored as <5% (1+), 5–50% (2+), and >50% (3+). Inflammation was scored as mild (1+) and extensive (2+). Psammoma bodies were absent (0) and present (1+). These grading schemes were done on haematoxylin and eosin slides of tumour and grading cutoffs were chosen from the previous studies [4, 7, 8]. All parameters used were easily reproducible, requiring no special techniques.

Statistical analyses were done using Fleiss kappa for intraobserver agreement between three pathologists for five parameters and kappa value was calculated. Fleiss kappa works for any number of raters giving categorical ratings to a fixed number of parameters. Kappa value interpretation was done according to Landis and Koch (1977) interpretation table as follows: ≥0 poor, 0.01–0.20 slight, 0.21–0.40 fair, 0.41–0.60 moderate, 0.61–0.80 substantial, and 0.81–1.00 almost perfect agreement.

## 3. Results

**Figure 1:**
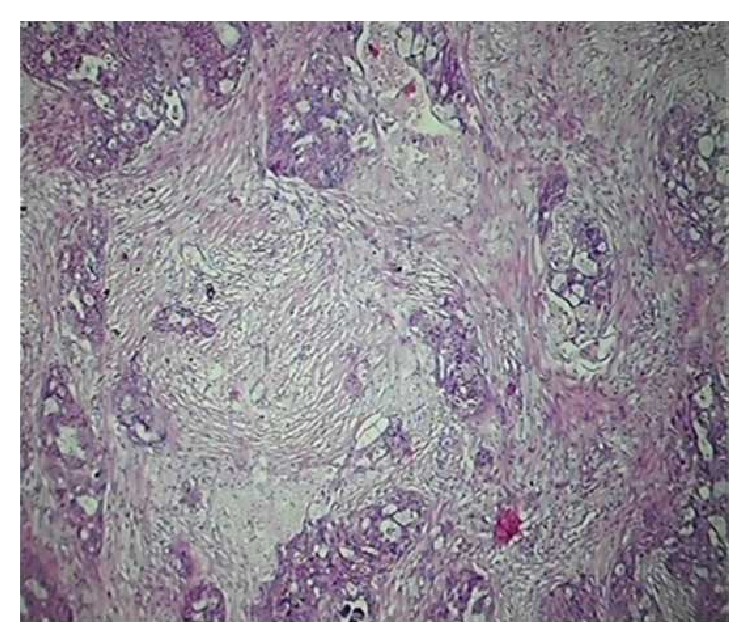
Sections showing moderate fibrosis (H & E ×100).

**Figure 2:**
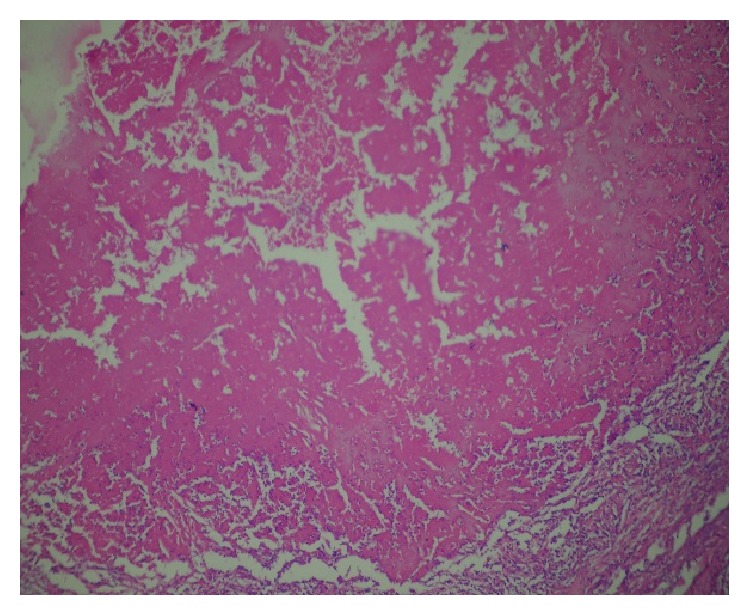
Sections showing necrosis (H & E ×100).

**Figure 3:**
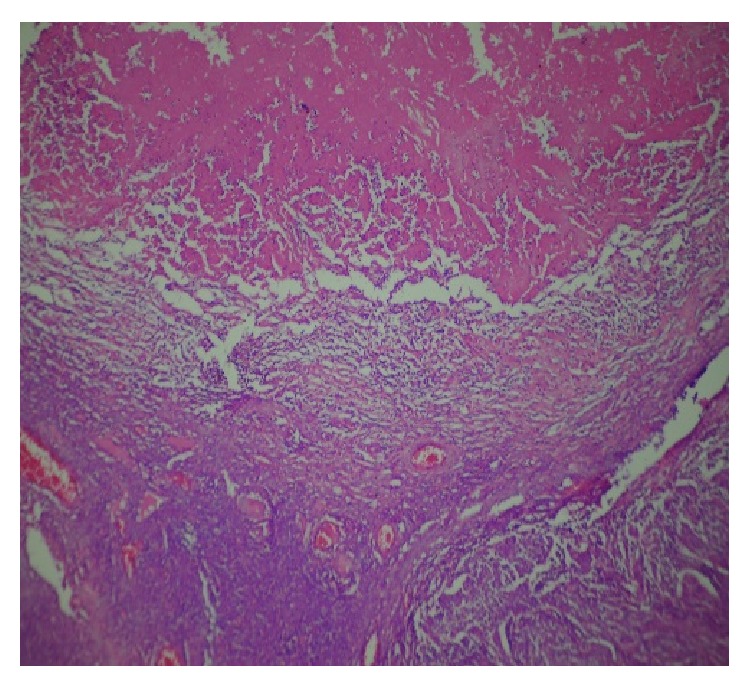
Sections showing extensive inflammation (H & E ×100).

**Figure 4:**
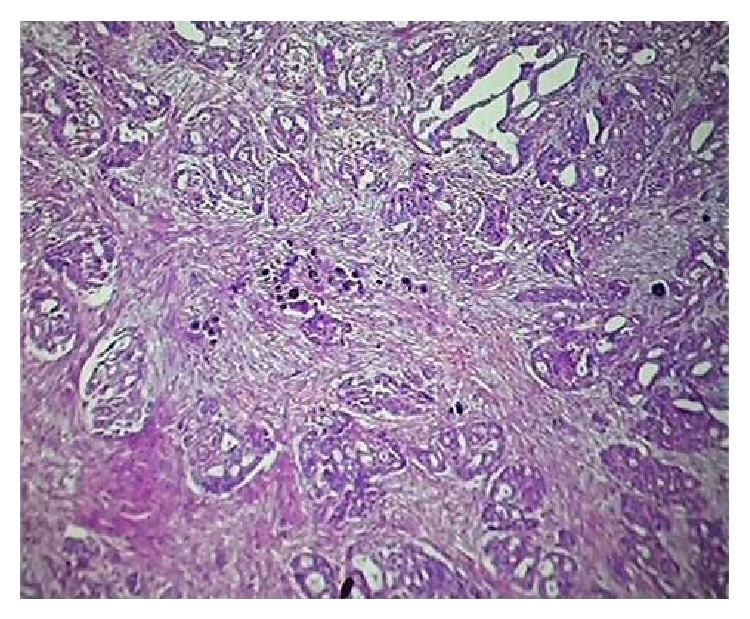
Sections showing residual tumour along with psammoma bodies (H & E ×100).

The present study is a prospective study of 40 cases. The patients age ranged from 36 to 82 years and mean was 50.7 ± 9.9 years. All these 40 cases were selected according to three inclusion criteria, that is, Ultrasound guided FNAC (fine needle aspiration cytology) proven, raised CA125 and those having malignant effusion. Patients having any one of the abovementioned or combination of any of these three were chosen. In this study five histological parameters were studied and summarized in Table 1. Moderate fibrosis (2+) was seen in 24 (60%) cases. 24 (60%) cases had necrosis (1+) whereas residual tumour was seen in 36 (90%) cases but it was less than 5% (1+). Extensive inflammation (2+) was present in 32 (80%) cases and psammoma bodies were present in 15 (37.5%) of all cases (Figures 1, 2, 3, and 4).

Overall intraobserver agreement in all five parameters highlighted substantial to almost perfect agreement. In three parameters, that is, fibrosis, inflammation, and psammoma bodies, intraobserver agreement was substantial with kappa values being 0.655, 0.621, and 0.776, respectively. Almost perfect agreement was seen in case of residual tumour and necrosis having *κ* values of 1.00 and 0.804, respectively.

## 4. Discussion

Neoadjuvant chemotherapy is an upcoming treatment modality for ovarian carcinomas [9]. However the morphological changes after neoadjuvant chemotherapy have not been described in detail. There is definitive need for the pathologists to understand and assess these histopathological changes as difficulty in tumour typing, grading, and identification of residual tumour arises [10].

There have been very few studies done to address this issue. Only one study done by Samrao et al. has objectively evaluated these morphological parameters [4]. They had largest series of 67 patients and the histological parameters chosen were easily reproducible, whereas in comparison the other studies had smaller case series of 18 patients and the parameters chosen included volume percentage of epithelium, mean nuclear area, and clinical response to chemotherapy which were difficult to reproduce [11]. This study had comparatively larger number of patients and in comparison to Samrao et al. number of parameters was increased to five by inclusion of psammoma bodies [4]. According to study done by McCluggage et al. presence of psammoma bodies without residual tumour was considered good response to chemotherapy [10].

The statistical analysis of intraobserver agreement to define reproducibility of parameters among pathologists each evaluating five sets of morphological changes showed an almost perfect agreement (*κ* range of 0.62–1.00). These findings were in concordance with those of Samrao et al. (*κ* value of 0.65–0.97) [4]. The most important factor was that the chosen histopathological criteria were easily reproducible and they do not require much experience or any ancillary aids for assessment.

Neoadjuvant therapy is an upcoming treatment modality and various studies have documented morphological changes induced by therapy in sites like colon, breast, esophagus, and lungs [12–14]. However not much literature is available on neoadjuvant chemotherapy induced histopathological changes of ovarian cancer. This leads to erroneous diagnosis particularly when history of chemotherapy is not clearly stated [15]. On the basis of guidelines published by CAP (College of American Pathologists) recent American Joint Committee on Cancer (AJCC) staging manual has recommended change in reporting format with stress on morphological changes induced by chemotherapy in colorectal carcinoma [16]. Authors want to stress upon the fact that such changes should be done in ovarian carcinoma formats.

In present study the tumour grading and subtyping were not done. Both are considered of prognostic value. Authors want to stress upon the fact that neoadjuvant chemotherapy induced changes make such evaluation impossible which has been confirmed by various studies [10, 11]. All prognostic markers become insignificant after chemotherapy so there is need to change reporting formats and our study wants to stress upon postchemotherapy changes and make pathologists aware of such morphological changes. All the parameters considered in this study are easily reproducible and do not require much expertise on part of pathologists and their inclusion in final histopathological report would help in patient management.

## Figures and Tables

**Table 1:** Distribution of five histological parameters.

Histological parameter	Grading		
Fibrosis	1+	2+	3+
	8 (20%)	24 (60%)	8 (20%)

Necrosis	0	1+	2+
	15 (37.5%)	24 (60%)	1 (2.5%)

Residual tumour	1+	2+	3+
	36 (90%)	4 (10%)	0 (0%)

Inflammation	1+	2+	
	8 (20%)	32 (80%)	

Psammoma bodies	0	1+	
	25 (62.5%)	15 (37.5%)	
